# Determination of standard number, size and weight of mediastinal lymph nodes in postmortem examinations: reflection on lung cancer surgery

**DOI:** 10.1186/1749-8090-8-94

**Published:** 2013-04-16

**Authors:** Sedat Ziyade, Nur Buyuk Pinarbasili, Nihan Ziyade, Osman Cemil Akdemir, Feyzi Sahin, Ömer Soysal, Alper Toker

**Affiliations:** 1 Department of Thoracic Surgery, Faculty of Medicine, Bezmialem Vakif University, Merkezefendi Mah. Mevlana Cad. Sedeftepe Evleri No:96/15 Zeytinburnu, İstanbul, Turkey; 2 Department of Pathology, Faculty of Medicine, Bezmialem Vakif University, İstanbul, Turkey; 3 The Ministry of Justice Council of Forensic Medicine Postmortem Microbiology Laboratory, İstanbul, Turkey; 4 The Ministry of Justice Council of Forensic Medicine, İstanbul, Turkey; 5 Department of Thoracic Surgery, Istanbul Medical School, Istanbul University, İstanbul, Turkey

**Keywords:** Lung cancer, Mediastinum, Lymph node, Dissection, Autopsy

## Abstract

**Background:**

Mediastinal lymph node dissection is an essential component of lung cancer surgery. Literature lacks established information regarding the number and size of the healthy lymph nodes. In this postmortem autopsy study, we aim to define the number, size and weight of the lymph nodes in each mediastinal lymph node station. To implement the data for the clinical practice, we analyzed the possible number of nodes to be dissected in a systematic mediastinal lymph node dissection from the right and left sides during lung cancer surgery.

**Methods:**

Sixty-two samples obtained from cadavers who did not die from chest malignancies, extrathoracic malignancies, any kind of infections or previous hospitalization before the death were included to the study. The locations of the nodes were recorded according to the American Thoracic Society Mediastinal Lymph Node Map. The number, size and weight of the nodes were determined at each station.

**Results:**

Median age of the cadavers was 39 years. Primary causes of death were asphyxia in 10 (16.1%) subjects, trauma in 29 (46.8%) subjects, cardiovascular problems in 10 (16.1%) subjects, and undetermined in 13 (21%) subjects. The median number of lymph nodes resected from each patient was 23 (range: 11–54). The right sided paratracheal lymph nodes (Station 2R and 4R) were more frequent, heavier and longer than left sided lymph nodes (Station 2L and 4L) at the paratrecheal region. Right sided inferior mediastinal lymph nodes were heavier and longer than the left ones; however, their availability was more often on the left.

**Conclusions:**

The properties of mediastinal lymph nodes at particular stations are different for number, size and weight. Station 4R and 7 have the highest number of nodes followed by stations 5 and 6. We recommend removing the lymph nodes of these stations completely in lung cancer patients to rule out the possibility of micrometastatic disease. Diameter of normal lymph node may be 1 cm for the stations other than 4R and 7, but the definition of normal diameter of a lymph node at the stations 4R and 7 may be changed as 1,5 cm and 2,0 cm, respectively. Weight of the nodes may be a new subject to study and may be defined as a new modality to define a staging to be more accurate and the issue needs further investigations.

## Background

The debate on the extent of mediastinal lymph node dissection (MLND) and its possible effect on prognosis of patients who undergo lung resection for lung cancer remain ongoing. With increasing evidence of importance of the accurate mediastinal lymphatic staging, thoracic surgeons try to standardize the terminology, definitions and their procedures [1]. It is accepted that, without MLND or systematic mediastinal lymph node sampling (MLNS), an accurate staging will be impossible. Indications for radical mediastinal lymphadenectomy remain unclear with controversy regarding its benefits and safety. Proponents of radical mediastinal lymphadenectomies (RML) claim that the procedure is safe, that it improves accuracy of staging and provides survival benefit [2,3]. Opponents of mediastinal dissection were concerned about the increased length of hospital stay secondary to increased morbidity, blood loss, duration of the intervention, higher incidence of recurrent laryngeal and phrenic nerve injuries, chylothorax and bronchopleural fistula [4]. In the literature, there are only two randomized studies which state that these concerns are unfounded [3,5]; however, micrometastatic disease is diagnosed more accurately with the method [2,4] and in various nonrandomized comparative studies a survival benefit for MLND has been clearly shown [2,3]. Despite the extensive effort for mediastinal lymph node evaluation in cancer patients by number, size and weight, normal mediastinal lymph nodes in healthy subjects have not been accounted for so far. We hypothesized that the number and size of lymph nodes may be different in each particular lymph node station and standardization of lymph node dissection or sampling may require consideration on the original number, size and weight in the otherwise healthy, postmortem subjects. In this autopsy study, we aim to define the number, size and weight of the lymph nodes in each mediastinal lymph node station.

## Methods

### Subjects

**Figure 1:**
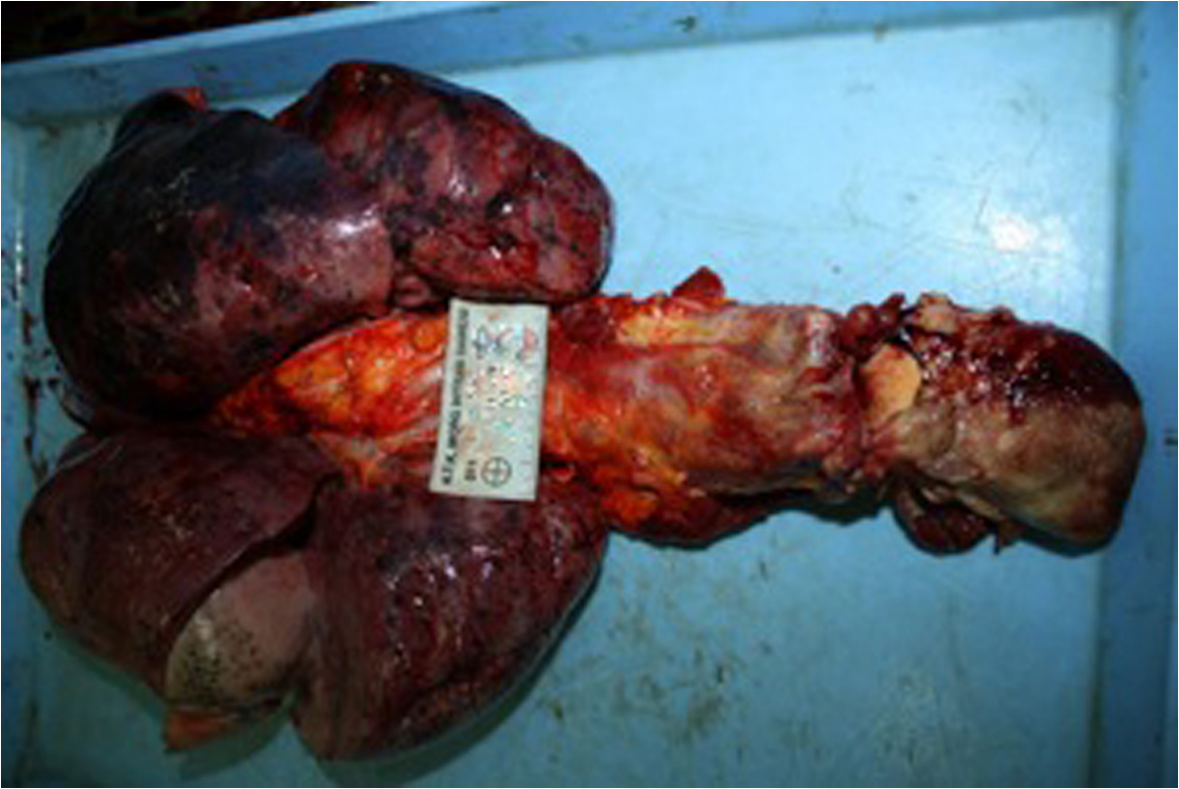
The mediastinum and lung were resected en bloc.

**Figure 2:**
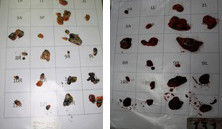
Separation and labelling of the nodes; two separate cases.

**Figure 3:**
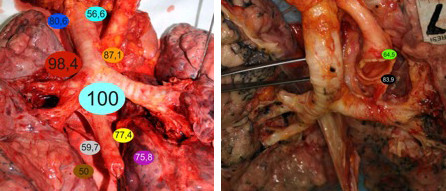
Lung after dissection of the mediastinal nodes with lymph node precence rate (eg.4R; 98,4%).

This study was conducted at the Istanbul Forensic Science Laboratory between January 2011 and June 2011 and study approved Istanbul Forensic Science Laboratory Scientific Study Council by the number B.03.1.ATK.0.01.00.08/27. The study population was selected from the cadavers who were sent to this center for legal autopsy. Sixty-four cadavers were included in the study. They did not have any conditions that might affect the size, weight or number of mediastinal lymph nodes such as chest malignancies, extrathoracic malignancies, any kind of infections or previous hospitalization before death. The dissection was completed within the first 24 hours after exitus. The mediastinum and lungs were resected en bloc from soft palate to diaphragm by resecting soft palate, tongue, esophagus, larynx, trachea and the part of aorta just above the diaphragm (Figure 1). Anatomic locations of the mediastinal lymph nodes were recorded according to the American Thoracic Society Mediastinal Lymph Node Map [6]. Lymph nodes dissections were done in order of station 9 to the station 2 bilaterally by an expert thoracic surgeon by removing the fat surrounding the each lymph node (Figure 2 and 3). The number, size (long axis of the lymph node in mm) and weight (in grams as weighted by Radwag as 220/C2 Analytic Scale®) of the lymph nodes were determined for each station of mediastinum. The lymph nodes were placed separately in boxes with 37% formalin for further pathological examination and one of the resected hilar lymph node during the mediastinal lymph nodes resection was sent for microbiological analysis. Besides the resected lymph nodes, the whole parts of the resected lung were also sent for pathological and microbiological examinations. The case was excluded from the study population when any infections were detected in those lung tissues. All dissections and separations were carried out by the same thoracic surgeon (S.Z.).

### Microbiological analysis

The microbiological examinations were performed at postmortem microbiology laboratory of the Turkish Republic Ministry of Justice Forensic Medicine by same microbiologist (N.Z.). Lymph node samples were examined especially for tuberculosis and all of them underwent homogenization and decontamination with N-acetyl-L-Cystein-NaOH. Then, These 62 samples were inoculated in Lowenstein Jensen medium and evaluated by Ehrlich Ziehl Nielsen (EZN) staining technique. The subjects were excluded when any culture resulted as positive.

### Pathological analysis

The pathological examinations were performed at the pathology laboratory of Bezmialem Vakif University Faculty of Medicine by the same pathologist (N.B.P.). Formalin fixed lymph nodes were measured and numbers were recorded. They were sectioned in the longitudinal axis with 0.5 cm and embedded in paraffin blocks. Two-micron slices were stained with Hematoxylene Eosin and examined under the light microscope. Any histopathological situations that may cause lymphadenopathy (e.g. sarcoidosis, tuberculosis, lymphoma etc.) were excluded.

### Statistical analysis

Data were analyzed with the SPSS software version 19.0 for Windows (SPSS Inc., Chicago, IL, USA) for descriptive statistics and frequencies. All the values were presented as median minimum and maximum values or/and mean ± standard deviation. Odds ratio was presented as OD with 95% confidence limits. Qualitative data were evaluated with Pearson Chi square or Fischer’s Exact Tests. Quantitative data were examined with Independent Samples T test. Statistical significance was defined if p value was <0,05.

## Results

During the study period 64 cadavers were included in the study but two cadavers were excluded from the study because post-mortem examination revealed tuberculosis in one of them and lung infection in the other. A total of 62 cadavers were evaluated with the age median of 39 years (min 12 years – max 79 years). Primary causes of death in our study population were trauma in 29 (46.8%) subjects, asphyxia in 10 (16.1%) subjects, cardiovascular problems in 10 (16.1%) subjects, and undetermined in 13 (21%) subjects.

From mediastinal 2R, 2L, 3A, 3P, 4R, 4L, 5, 6, 7, 8R, 8L, 9R and 9L lymph node stations (total 13 stations) a mean of 9,9 stations were found to have a tissue to be dissected. Out of these a median of 1 (min:0 - max:3) lymph node stations were found to be false positive due to presence of fatty tissue, thymic tissue or muscle tissue instead of lymph node by light microscopy. All the false positive stations were excluded. The excluded false positive lymph nodes and the rate of lymph node positivity with respect to the station are shown on Table 1.

**Table 1:** An overview of the characteristics of the right and left sided mediastinal lymph node stations

**Lymph node stations**	**Excluded (false lymph node) (%) p**	**Node presence rate (%) P**	Weight of LN (gr) Median (min-max) p***	Longest diameter (mm) Median (min-max) p***	Number of LN Median (min-max) p***
2R-2L	11.3 – 6.5 n.s.*	80.6 – 56.6 0,004*	0,21 (0,02 - 3,26) 0,17 (0,01 - 1,16) n.s.	10 (4–30) 9 (3–20) **0,032**	2 (1-7) 2 (1–9) n.s.
4R-4L	0 – 8.1 n.s.**	98.4- 87.1 0,032**	1,08 (0,07 - 12,36) 0,36 (0,03 - 2,11) **0,000**	18 (5–44) 12 ( 4–31) **0,000**	4 (1–10) 2 (1–11) **0,000**
3A-3P	1.6-0 n.s.**	45.2 – 14.5 0,000*	0,21 (0,05 - 2,60) 0,11 (0,02 - 0,96) n.s.	10 (6–23) 6 (4–24) n.s.	1 (1–5) 1 (1–5) n.s.
5	1.6	83.9	0,50 (0,07 - 2,48)	13 (6–30)	3 (1–7)
6	16.1	64.5	0,30 (0,01 - 5,19)	10 (3–32)	3 (1–8)
7	0	100	2,12 (0,16 - 8,68)	27 (9–62)	4 (1–18)
8R-8L	16.1 – 9.7 n.s.*	59.7 – 77.4 0,003*	0,24 (0,03 - 3,40) 0,28 (0,04 - 35,0) n.s.	11 (4–29) 12 (2–26) n.s.	2 (1–5) 2 (1–6) n.s.
9R-9L	19.4-8.1 n.s.*	50-75.8 0,003*	0,19 (0,04 - 2,0) 0,25 (0,04 - 2,05) n.s.	9 (4–29) 12 (5–31) n.s.	2 (1–8) 2 (1–5) n.s

The heaviest, longest diameter and highest number zones are stations 4R and 7.

*Pearson Chi-Square Test, **Fisher’s Exact Test, *** İndependent samples T test, *R*: right, *L*: left, *LN*: Lymph node.

**Figure 4:**
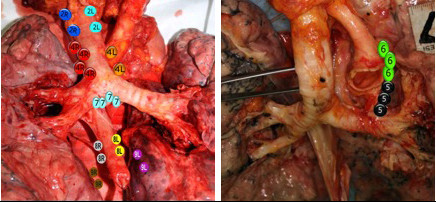
Lung after dissection of the mediastinal nodes with mean number of LN (eg. 4R; 4 LNs, mean).

The percentage of lymph node presence, the median of lymph node size, the median of weight of lymph nodes and the median number of mediastinal lymph node with respect to stations were summarized as follows and presented in Figure 3 and 4.

### Station 2 lymph nodes

The availability frequency of the lymph nodes (LN) at Station 2R was 80,6% whereas 56,6% at the corresponding site (p:0,004) with rough inspection among the cadavers. However, after comprehensive histopathologic dissection we detected median number of 2 LN at each site (p:0,33). There was not a statistically significant difference between the median weight of LN (gr) at Station 2R and Station 2L [0,21 gr. at 2R vs. 0,17 gr. at 2L. (p: 0,06)]. The median longest lymph node diameter (mm) was statistically different between the stations 2R and 2L, as 10 mm and 9 mm, respectively (p: 0,03).

### Station 4 lymph nodes

The detailed histopathological examination of the specimens at the station 4R and 4L revealed 4 and 2 LN (p:0,000), respectively, although we resected LN at 98,4% rate from Station 4R and 87,1% from 4L (p:0,032) from the en bloc tissues. The median weight of resected LN tissues were 1,08gr vs. 0,36gr on the right and the left sides (p:0,000). The lengths of the LN were measured median of 18 mm on the right side and 12 mm on the left side (p:0,000).

The stations 2 and 4 are paratracheal regions and located at each side of the trachea. The right sided paratracheal LN were more frequent, heavier and longer than left sided LN at the paratrecheal region.

### Station 3 lymph nodes (3 anterior and posterior LN)

Station 3 LN are located at the anterior and posterior regions of the trachea rather than right and left side. The presence of LN at the anterior portion was %45.2 and %14.5 posteriorly among the cadavers, (p:0,000). Histopathological dissection revealed 1 LN at each side The median weight was 0,21gr at Station 3A and 0,11gr at Station 3B (p:0,29). The median LN diameters were 10 mm and 6 mm at 3A and 3P, respectively (p:0,31).

### Station 5 and 6 lymph nodes

Station 5 and 6 LN are perivascular LN and located only at the left side of the mediastinum near the aorta and the pulmonary artery. They were detected at a frequency of 83,95 at Station 5 and 64.5% at Station 6; however, histopathological availability was 3 nodes at each. The corresponding median weights were 0,50gr and 0,30gr and diameters were 13 and 10 mm.

### Station 7 lymph nodes

Station 7 is located below the carina and regarded as the central LN station. The lymph nodes were 100% available in all patients during dissection with a median number of 4. Moreover, the Station 7 included the heaviest and longest LN among the population with a median weight of 2,12gr and diameter of 27 mm, respectively.

### Station 8 lymph nodes

The presence of LN among the specimens were 59.7% vs. %77.4% at the right and left sides, respectively (p:0,033). Dissections revealed 2 LN at each side (p:0,37). There was not a statistically significant difference between the weight (0,24 at 8R vs. 0,28 at 8L, p:0,37) and length (11 mm at 8R and 12 mm at 8L, p:0,44).

### Station 9 lymph nodes

The detailed histopathologic examination of the specimens at the Station 9R and 9L revealed 2 and 2 LN (p:0,97), respectively although we could resect LN at 50% rate from Station 8R and 75.8% from 8L (p:0,003) from the cadavers. The median weight of resected LN tissues were 0,19gr vs. 0,25gr at the right and the left sides (p:0,32). The lengths were measured median of 9 mm at Station 8R and 12 mm at the Station 8L (p:0,09).

*Although statistical analysis did not reveal a statistically significant difference between the weight and length of the LNs at the inferior mediastinal LNs, i.e., Station 8 and 9, the diameters and weight were higher at the left sided LNs. Additionally, although the histopathologic dissections of each specimen from Station 8 and 9 revealed 2 LNs on each side, their availability was more often on the left side in the population* (Table 2)*.*

**Table 2:** Comparison of left and right sided lymph nodes

**Total**	**Group**	**N**	**Median (min-max)**	**Mean**	**Std. Deviation**	**P**
Weight	R	179	0,41 (0,02-2,36)	0,90	1,39	n.s.
	L	277	0,35 (0,01-35)	0,66	2,15	
Length	R	179	13 (4–44)	14,16	7,41	**0,045**
	L	277	12 (2–32)	12,85	5,68	
Number	R	179	2 (1–10)	2,92	1,95	**0,010**
	L	277	2 (1–11)	2,48	1,56	

Left and right sided nodes are compared. Stations 3A, 3P and 7 are excluded and stations 5 and 6 nodes are included in the left sided nodes. Lymph nodes were equal in weight, however, right sided nodes are demonstrated to be larger in diameter and higher in number (Independent samples T test).

The demographic features of each lymph node station are presented on Table 1.

### Right sided stations vs. left sided station

Right sided stations were present for Stations 2, 4, 8, and 9 whereas left ones included Stations 2, 4, 5, 6, 8, and 9. When assuming that the total number of expected LN to be at least 248 (4 lymph node stations in 62 cadavers) in the total, we were able to detect 179 (72.1%) LN on the right side. In contrast, the expected number of LN was to be at least 372 for the left side; however, we dissected 277 (74.4%) LN from the left side. There was a statistically insignificant difference between the number of the LN at each side (p:0,748). The mean of the weight of right sided LN were 0,90±1,39gr and 0,66±2,15 gr. for the left side which was not statistically significant (p:0,45 ). On the other hand, mean of the diameter of the LN was significantly different between sides as 14,16±7,41 mm on the right side vs. 12,85±5,68 mm on the left (p: 0,045). Histopathologic dissection revealed a mean number of 2,92±1,95 LN on the right side and 2,48±1,56 LN on the left side (p:0,010). As a result, weight of lymph nodes with respect to side of localization were not different at each side. The right sided lymph nodes were found to be longer in diameter and more in number than left sided ones (p<0.05) [Table 2].

In implementation of this data to the clinical practice, we have analyzed the possible number of nodes to be dissected during a systematic mediastinal lymph node assessment with respect to side. We compared the stations 2R, 4R, 7, 8, and 9 of the right side to stations with 5, 6, 7, 8, and 9 of the left side. We found out that 12 lymph nodes should be dissected on right systematic MLND and 11 lymph nodes would be dissected on left systematic MLND. Thus, systematic mediastinal nodal dissection on both sides demonstrated statistically indifferent number of nodes (Table 3).

**Table 3:** Systematic dissection in right and left thoracotomies

	**Median (n)**	**Mean (n)**	**S.D.**	**p**
Right systematic MLND	12 nodes (4–30)	12.5	4.5	n.s.
Left systematic MLND	11 nodes (3–24)	11.4	4.9	

Independent samples T Test.

## Discussion

Despite highly developed noninvasive and invasive techniques like PET-CT, mediastinoscopy, EBUS or EUS, preoperative accurate diagnosis of mediastinal lymph node metastasis is still a problem for thoracic surgeons as well as patients undergoing surgery for lung cancer. Therefore, intraoperative mediastinal node dissection is still the gold standard for pathological staging of thoracic malignancies [1]. Standardized techniques of mediastinal nodal dissection were recommended based on the data retrieved from lung cancer patients without implementing the findings of normal subjects. Lymph nodes detected in lung cancer patients may demonstrate physical properties not only due to metastasis but also due to underlying pneumonia. We believed a revisit is required on normal size, weight and length of the mediastinal lymph nodes in healthy subjects due to lack of data to help the standardization of the surgical procedures for intrathoracic tumors. Literature lacks an established study for mediastinal lymph node dissection of healthy subjects. Only seldom reports mention about autopsy and computerized chest tomography findings [7]. The current study gives information for the first time in the literature about features of mediastinal lymph nodes, which may be taken as normal in autopsy series. For instance, we found the largest and normal mediastinal nodes in the right paratracheal and subcarinal regions and, the right sided nodes are larger than the left sided nodes.

This lymph node evaluation study was done in otherwise normal healthy subjects died from noninfectious and non oncologic reasons. Paratracheal lymph node dissection is a general practice during lung cancer surgery in the right thoracotomy; however, left sided paratracheal dissection needs additional maneuvers and commonly not employed in the routine practice. In this study, we have demonstrated that right sided nodes, the 2R and 4R, are more commonly present than their counterparts. Station 4R is demonstrated to be longer in diameter and heavier in weight than station 4L. Additionally, station 7 has been shown to be the highest rate of node presence (100%), the heaviest (2,12 gram median), and the longest (27 mm median) one among the stations. In the inferior mediastinal node evaluation, number of lymph nodes was demonstrated to be higher in the left side when we compared stations 8 and 9; however, their length, weight and number were similar in both sides. In comparison of the two hemithoracic dissections, lymph nodes were shown to be equal in weight; however, right sided nodes are demonstrated to be larger in diameter and higher in number. In implementation of this data into clinical practice, we have analyzed the possible number of nodes to be dissected in a systematic mediastinal lymph node dissection in right and left sides by comparing the stations 2R, 4R, 7R, 8R, 9R stations and 5L, 6L, 7L, 8L, 9L stations. Systematic dissection revealed almost equal number of nodes in both sides. This study showed that a median of 12 nodes in the right and 11 nodes in the left chest have been resected from the mediastinum in healthy subjects; however, literature concerning mediastinal nodal dissection reveals discrepancy, creating a real confusion for chest surgeons. Excised number of mediastinal nodes with SND have been presented to be 17.3 nodes [3], 38.9 nodes (bilateral mediastinal dissection with transcervical technique, excluding station 9) [8], 40.3 in the right vs. 37.1 in the left (including hilar resion and stations 11 and 12) [9], 8.6 nodes (with video assisted mediastinoscopic lymphadenectomy (VAMLA) from paratracheal stations and station 7 [10], 16 nodes with VAMLA [11], and 8.4 nodes from paratracheal region only via left thoracotomy [12]. The presence of either micro metastasis, reaction to a tumor or to an infection in the lung distal to the tumor may be the reason for these discrepancies.

In this study we have shown that stations 4R and 7 have a median of 4 nodes and removing single of node from these stations may not totally reflect the micrometastatic disease located in these stations, where as removing a single node from station 2R, 8R, 9R may have a higher possibility to reflect the actual situation due to presence of median number of 2, 2, 2 nodes, respectively. Similarly in the left hemithoracic stations 5 and 6 have a median of 3 nodes. Thus removing a single node from these stations may not reflect the actual pathological situation as removing a single node from station 8 and 9 which have a median of 2 nodes. As a result one can recommend removing more nodes compared to other stations or perform a complete dissection of the stations 4R and 7 in the right hemithorax and stations 5, 6 and 7 in the left chest. In the analysis of imaginary right and left sided systematic SND, we have demonstrated that a median of 12 and 11 nodes were removed, which merits almost the similar number proposed by Goldstraw [13], which is a recommendation of 10 nodes from 3 stations including station 7. According to the results of the current study similar sampling from each station may not be the correct approach. For example, sampling of a single node from station 2R, 8R and 9R have the possibility of 50% to find a micrometastatic disease whereas this possibility decreases to 25% in stations 4R and 7. To increase reliability of tumor free rate up to at least 50% during oncologic surgery, surgeons have to remove at least 2 nodes from stations 4R and 7.

In addition to the variability of the number of lymph nodes, the size was demonstrated to be longer in stations 4R and 7. In the study, we have calculated the median longest diameter as 11 mm. However, in stations 4R and 7, lymph nodes as big as 18 mm and 27 mm, respectively, were also detected found. This findings showed similarities with previous papers [7,14]. Stations other than 4R and 7 revealed lymph nodes with median longest diameter of 10 mm. This may be a useful data for a developing country or immigrant patients in developed countries and may not reflect the actual size in the developed country populations. Additionally, the information may prevent to perform unnecessary diagnostic mediastinoscopies in healthy patients with enlarged 4R and 7 stations with negative positron emission computerized tomography (PET-CT) findings. We may define the normal lymph node diameter for different stations. Normal lymph node diameter may be 1 cm for the stations other than 4R and 7. The new definition of normal diameter of 4R and 7 may be 1.5 cm and 2,0 cm, respectively.

We have studied weight of the lymph nodes in each station with a very sensitive scale. It was shown that stations 4R and 7 are the heaviest stations. Currently, the weight of a lymph node does not have any clinical implication, but in the very near future it seems a new staging modality may bring the weight of lymph node as an indication for completeness of dissection. Recently, an article comparing VATS with open lung resections with mediastinal lymph node dissection used this method [9]. The data may be a new insight for the researchers. Weighing the lymph nodes prior to formalin fixation and right after dissection may be recommended.

### Study limitations

The cadavers are confined to a specific geographic location and can not represent the lymphatic characteristics of the world population, which may be accounted as a limitation. Another limitation is the median age, which is relatively smaller than the lung cancer population. Smoking history could not been obtained since it was not ethically correct to talk to the families of such victims about smoking history. We do not think that population being healthy subjects is not a limitation. As we have mentioned before, there were a lot of studies in lung cancer populations, and our primary aim was to define the situation in non malignancy and infection cases, and only to obtain some information about normal anatomy of mediastinal lymph nodes.

## Conclusions

We have 4 conclusions. 1- Mediastinal lymph node stations have different properties of the number, size and weight. Station 4R and 7 have the highest number of nodes followed by stations 5 and 6. We recommend removing these stations completely or removing more lymph nodes from these stations to rule out the possibility of a micrometastatic disease. 2- Normal lymph node diameter may be 1 cm for the stations other than 4R and 7. The new definition of normal diameter of 4R and 7 may be 1.5 cm and 2,0 cm, respectively. 3- Twelve nodes in the right and 11 nodes in the left chest should be resected for lung cancer systematic lymph node dissection; 4- Weight of the nodes may be a new subject to study and may be defined as a new modality to define a staging to be more accurate and the issue needs further investigations.

## Competing interest

The authors declare that they have no competing interests.

## Authors’ contribution

SZ designed the study and written the manuscript, OCA, FS and NZ applied the autopsies, NB applied the pathological examinations, AT participated in the design of the study. ÖS participated the study design, coordination and helped to draft the manuscript. All authors read and approved the final manuscript.
